# Kinetic Insights into Methanol Synthesis from CO_2_ Hydrogenation at Atmospheric Pressure over Intermetallic Pd_2_Ga Catalyst

**DOI:** 10.1002/gch2.202400159

**Published:** 2024-09-20

**Authors:** Kaisar Ahmad, Aasif Asharafbhai Dabbawala, Kyriaki Polychronopoulou, Dalaver Anjum, Marko Gacesa, Maguy Abi Jaoude

**Affiliations:** ^1^ Center for Catalysis and Separation Department of Chemistry Khalifa University of Science and Technology PO Box 127788 Abu Dhabi UAE; ^2^ Center for Catalysis and Separation Department of Mechanical and Nuclear Engineering Khalifa University of Science and Technology PO Box 127788 Abu Dhabi UAE; ^3^ Center for Catalysis and Separation Department of Physics Khalifa University of Science and Technology PO Box 127788 Abu Dhabi UAE

**Keywords:** alternative fuels, CO_2_ utilization, intermetallic effect, microkinetic modeling, reaction path analysis

## Abstract

This study presents a single‐site microkinetic model for methanol synthesis by CO_2_ hydrogenation over intermetallic Pd_2_Ga/SiO_2_. A reaction path analysis (RPA) combining theoretical results and realistic catalyst surface reaction data is established to elucidate the reaction mechanism and kinetic models of CO_2_ hydrogenation to methanol and CO. The RPA leads to the derivation of rate expressions for both reactions without presumptions about the most abundant reactive intermediate (MARI) and rate‐determining step (rds). The formation of H_2_COOH* is found to be the rds (step 19) for methanol synthesis via the formate pathway, with CO_2_ and H‐atoms adsorbed on intermetallic sites as the MARIs. The derived kinetic model is corroborated with experimental data acquired under different reaction conditions, using a lab‐scale fixed‐bed reactor and Pd_2_Ga/SiO_2_ nanoparticles prepared by incipient wetness impregnation. The excellent agreement between the experimental data and the kinetic model (*R* ^2^ = 0.99) substantiates the proposed mechanism with an activation energy of 61.52 kJ mol^‐1^ for methanol synthesis. The reported catalyst exhibits high selectivity to methanol (96%) at 1 bar, 150 °C, and H_2_/CO_2_ ratio of 3:1. These findings provide critical insights to optimize catalysts and processes targeting CO_2_ hydrogenation at atmospheric pressure and low temperatures for on‐demand energy production.

## Graphical abstract

A new microkinetic study integrates theory, experiments, and reaction pathways to explore CO₂ conversion to methanol over Pd_2_Ga/SiO_2_ catalyst. The CO_2_‐conversion is 96% selective to methanol at 150 °C under 1 bar pressure. The H₂COOH* formation is identified as the rate‐determining step with an activation energy of 61.52 kJ mol^−1^. The derived kinetic models and mechanism reliably predict the experimental data.

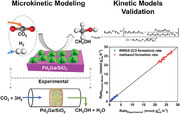

## 1 Introduction

The overwhelming global climate change impacts inflicted by carbon emissions due to the omnipresent fossil fuel economy demand an urgent paradigm shift to create a sustainable roadmap for the energy sector and dependent industries. Using carbon dioxide (CO_2_) captured from air or point emission sources to produce low‐carbon fuels (e.g., syngas, blue methanol) and value‐added chemicals is a potentially fruitful path to achieving carbon neutrality. There has been significant interest in the utilization of renewable methanol for the production of sustainable aviation fuels (SAF) and as electrofuel for storing green hydrogen in a liquid state (CO_2_(g) + 3H_2_(g) → CH_3_OH(liq) + H_2_O(liq) in power‐to‐X (PtX) conversion technologies.^[^ ^1^ ^]^ The CO_2_ molecule with high formation energy needs a highly active, stable, and selective catalyst for methanol synthesis, to prevent the side formation reaction of CO via the reverse water gas shift (CO_2_ + H_2_ → CO + H_2_O).^[^ ^2^ ^]^ The industrial Cu/ZnO/Al_2_O_3_ catalyst traditionally used for methanol synthesis via CO exhibits low selectivity to methanol and deactivates readily in the direct hydrogenation of CO_2_ due to Cu‐sintering. Alternative catalyst formulations with similar or improved performance have been developed, including Cu/CeO_2_,^[^ ^3^ ^]^ Au/ZnO,^[^ ^4^ ^]^ Pd/Ga_2_O_3_,^[^ ^5^ ^]^ In_2_O_3_,^[^ ^6^ ^]^ and Pd/ZnO.^[^ ^7^ ^]^ Nonetheless, the reaction thermodynamics over these catalysts disfavor methanol synthesis due to the high operating temperatures required (>270 °C), leading to reduced CO_2_ conversion. Recently, intermetallic catalysts like Ni_5_Ga_3_^[^ ^8^ ^]^ and Pd_2_Ga^[^ ^9^ ^]^ have shown higher activity than Cu/ZnO/Al_2_O_3_ for methanol synthesis at atmospheric pressure (*P* = 1 bar). Also, a methanol selectivity >95% has been achieved at atmospheric pressure without any dealloying or deactivation of the catalysts. In a study, Quilis et al.^[^ ^10^ ^]^ found that an interface between intermetallic β‐PdZn and ZnO is highly active for methanol synthesis with a yield of 44.48 mmol_methanol_/hg_Pd_. Similarly, compared to their constituent single‐metal‐based catalysts,^[^ ^11^ ^]^ intermetallic compound catalysts exhibit superior activity, selectivity, and durability (e.g., resistance to sintering and coking) for methanol synthesis.^[^ ^8^, ^12^ ^]^ By alloying two metals in a specific composition (outside the solubility gap), a lower activation barrier can be achieved for a reaction to that over individual metals, under identical reaction conditions.^[^ ^13^ ^]^ The improved catalytic performance of intermetallics is attributed to their high density of active sites and synergy between the alloyed metals owing to geometric effects like active‐site isolation (ASI) and electronic effects. The geometric effect alters the shape and chemical environment of the area surrounding active sites, causing the latter to be structurally separated from one another. This results in increased stability and the possibility of performing selective reactions that are sensitive to structural changes, such as methanol synthesis from CO_2_ hydrogenation. In intermetallics, changing the coordination number of metal atoms causes electron transfer and hybridization, leading to enhanced interaction between the reactive gas medium and the intermetallic surface. Although ASI and electronic effects can happen simultaneously, the impact of the geometric effect on reaction selectivity is more significant than the incremental influence of charge transfer on surface interaction.^[^ ^14^ ^]^ For example, in a study on the Pd–Ag intermetallic catalyst, the charge transfer from Ag to Pd (d‐orbital) was found to decrease the binding energy of an alkyne by 10 to 20 kJ mol^−1^. At the same time, the ASI effect contributed to a 35 kJ mol^‐1^ reduction.^[^ ^15^ ^]^ This is because the latter differentiates and disrupts the correlation between the adsorption energies of the desired and undesired reaction intermediates, which has a profound impact on selectivity.^[^ ^16^ ^]^

Lowering the activation barrier of the reaction steps that lead to selective methanol production is a means by which intermetallic catalysts, like Pd_2_Ga, can achieve enhanced catalytic activity. The mechanism of methanol synthesis is complex, involving numerous direct and indirect pathways that include a variety of elementary reaction steps. The formation of methanol from CO_2_/H_2_ mainly starts via the formation of COOH*, HCOO*, and CO*, leading to a network of reactions, and the identification of the rate‐determining step (rds) is essential for determining the reaction mechanism.^[^ ^17^ ^]^ The hydrogenation of formate was previously established as rds over various catalysts like Cu(100),^[^ ^18^ ^]^ Cu/ZnO/K/Cr_2_O_3_,^[^ ^19^ ^]^ Cu/SiO_2_,^[^ ^20^ ^]^ Pd‐Cu‐Zn/SiC,^[^ ^21^ ^]^ CuO/CeO_2_/ZrO_2_,^[^ ^22^ ^]^ and Ga_3_Ni_5_ catalyst.^[^ ^23^ ^]^ Differently, Gao et al.^[^ ^24^ ^]^ found the hydrogenation of HCO as the critical step over the YBa_2_Cu_3_O_7_ catalyst via the carboxyl route. Similarly, Zhao et al.^[^ ^25^ ^]^ found that the trans‐COOH route, with the decomposition of COHOH* to COH* and OH* as the rds, is the most favorable pathway for methanol synthesis over Cu(111). In a similar study over intermetallic Ga_3_Ni_5_(111) catalyst, Tang et al.^[^ ^26^ ^]^ found the formation of H_2_O as the rds for the methanol synthesis through the carboxyl‐mediated pathway.

In this study, a novel approach was employed to correlate lab‐scale experimental data and density functional theory (DFT)‐calculated energies, aiming to unveil a reliable reaction mechanism and develop a kinetic model for the CO_2_ hydrogenation to methanol over a Pd_2_Ga catalyst using the reaction path analysis (RPA) technique. The experimental data were obtained by performing hydrogenation of CO_2_ to methanol over an intermetallic Pd_2_Ga/SiO_2_ catalyst, using a laboratory‐scale continuous fixed‐bed reactor. The proposed RPA approach aims to find the rds, most abundant reactive intermediate (MARI), and reaction pathways, establishing a kinetic model without presuming the rds and MARI. This is done even when mechanistic information is limited, contrasting with studies that typically assume and derive the kinetic model without prior identification of these parameters. From this study, gaining insights into the role of the intermetallic effect and the intrinsic kinetics of selective methanol synthesis at atmospheric pressure through the low‐energy reaction pathway on Pd_2_Ga/SiO_2_ will contribute to developing new and improved catalysts. This advancement aims to mitigate high‐pressure storage and transportation costs, ultimately establishing methanol synthesis units capable of utilizing captured CO_2_ at the point sources of green H_2_ production units.

## 2 Results and Discussion

### 2.1 High Resolution‐Transmission Electron Microscopy (HR‐TEM) and Powder X‐Ray Diffraction (p‐XRD)

**Figure 1:**
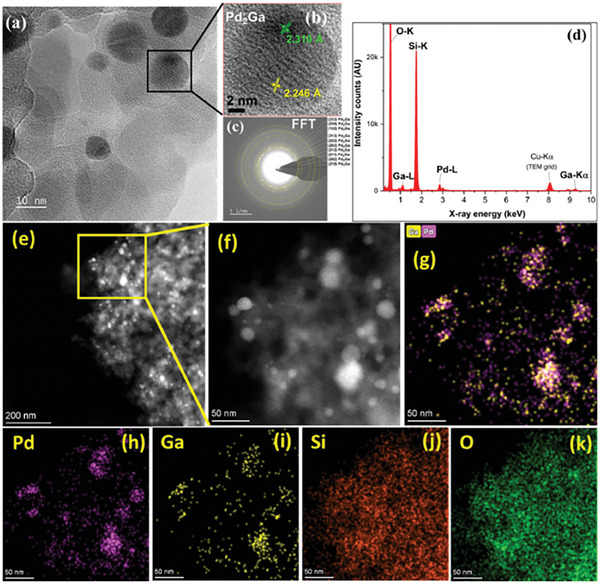
H_2_‐reduced Pd_2_Ga/SiO_2_: a,b) HR‐TEM images, c) selected area electron diffraction (SAED) pattern, d) TEM‐EDX, e,f) HAADF‐STEM micrograph, g–k) elemental color maps, magenta: Pd; yellow: Ga; red: Si; green: O.

The HR‐TEM images and corresponding Fast Fourier Transform (FFT) analysis of H_2_‐reduced catalyst nanoparticles (**Figure** **1**) evidence the presence of phase‐pure intermetallic Pd_2_Ga over the amorphous SiO_2_ support.

The HR‐TEM data and selected area electron diffraction (SAED) pattern (Figure 1b,c) evidence the exposure of different crystal planes corresponding to the Pd_2_Ga phase.^[^ ^9a^ ^]^ The complementary scanning transmission electron microscopy with energy dispersive X‐ray spectroscopy (STEM‐EDX) spectrum (Figure 1d) confirms the presence of all the constituent elements of the supported catalyst and phase purity. High‐angle annular dark‐field (HAADF)‐STEM images of the Pd_2_Ga/SiO_2_ catalyst with mapping are presented in Figure 1e–k. From the HAADF‐STEM and mapping, it can be confirmed that the Pd and Ga are uniformly distributed over support with high dispersion.

**Figure 2:**
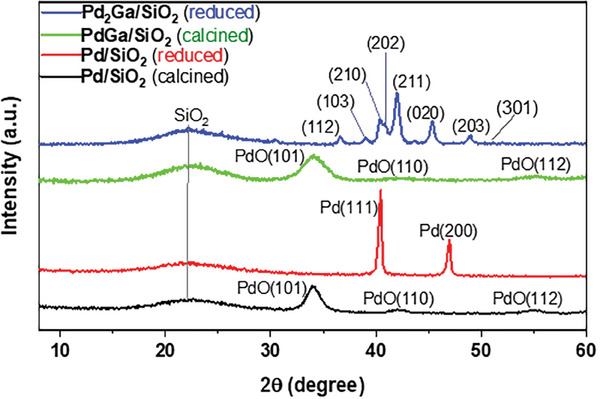
p‐XRD patterns of (reduced/calcined) Pd_2_Ga/SiO_2_ and Pd/SiO_2_ catalysts.

The p‐XRD patterns of Pd_2_Ga/SiO_2_ (calcined and reduced) and Pd/SiO_2_ (reduced) catalysts are plotted in **Figure** **2**. The diffraction peak pattern of the H_2_‐reduced Pd_2_Ga/SiO_2_ sample matches that of the phase pure Pd_2_Ga intermetallic compound (ICSD collection code 409939). The diffraction peaks corresponding with the 2‐theta values of 36.6°, 39.0°, 39.7°, 40.2°, 41.4°, 44.6°, 48.2°, 51.4° were indexed to the crystallographic planes (112), (103), (210), (202), (211), (020), (203), and (301), respectively.

Notably, metallic Ga or gallium oxide peaks were not observed in the obtained XRD patterns, suggesting that these phases are either non‐crystalline or exist in a liquid state. The high dispersion of Ga_2_O_3_ during calcination may be the reason behind the absence of Gallium species in the XRD patterns. Armbrüster et al.^[^ ^27^ ^]^ reported the reduction mechanism of Ga^3+^ species on Pd nanoparticles at higher temperatures, attributing it to the presence of highly active hydrogen atoms within palladium hydride. The XRD results agree with the SAED data with a crystal size of 5.73 nm corresponding to the (020) plane. The characteristic diffraction peaks of the intermetallic structure were absent in the pattern of the calcined sample, which exhibited only PdO peaks due to the amorphous nature of the silica support and gallium oxide. The absence of oxide peaks of PdO, in the reduced sample corroborates the formation of the Pd_2_Ga phase following its H_2_ reduction. In this study, a Pd/SiO_2_ sample in a reduced state was utilized for comparison and to clarify the influence of the intermetallic effect. The p‐XRD pattern of the control showed characteristic Pd phase peaks (ICSD collection code 648680) at 2‐theta values of 40.1° and 46.3° corresponding to the crystal planes (111) and (200), respectively.

### 2.2 Hydrogen Temperature‐Programmed Reduction (H_2_‐TPR)

**Figure 3:**
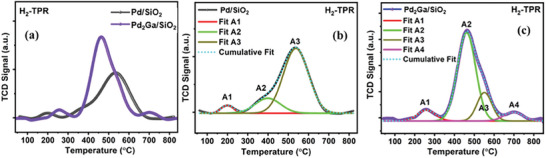
a) H_2_‐TPR profile of Pd/SiO_2_ and Pd_2_Ga/SiO_2_ catalysts. Deconvoluted profile for b) Pd/SiO_2_ and c) Pd_2_Ga/SiO_2_.

The temperature at which catalysts undergo reduction plays a critical role in determining their catalytic reactivity. The H_2_‐TPR technique was employed to assess the reducibility of the reported catalysts and gain insights into the interaction between the metal and the support material (**Figure** **3**). The H_2_‐TPR profile of Pd/SiO_2_ exhibited distinct peaks at 198 °C, 399 °C, and 538 °C, designated as A1, A2, and A3, respectively (Figure 3b). Among these peaks, A3 exhibited the highest intensity.

The reduction peak A1 observed at a lower temperature corresponds to the reduction of free or weakly bonded Pd oxide species on the silica support.^[^ ^28^ ^]^ Whereas the reduction peak A2, appearing between 300 °C and 500 °C, and the peak A3, observed above 500 °C, are attributed to the reduction of PdO species moderately and strongly interacting with the silica support, respectively. In the case of Pd_2_Ga/SiO_2_, the addition of Ga influences the reducibility of PdO. The reduction temperature of Ga_2_O_3_ is higher than that of PdO. The first peak A1 corresponds to the reduction of PdO, followed by the reduction of Ga_2_O_3_ and the alloying to form the Pd_2_Ga phase. The reduction peak appearing beyond 550 °C represents the alloying temperature that yields the Pd_2_Ga phase. Notably, the intensity of peak A2 in the H_2_‐TPR pattern of Pd_2_Ga/SiO_2_ is significantly higher than that of the Pd/SiO_2_ catalyst (Figure 3c). This occurs because the small Pd nanoparticles begin to alloy with Ga at 300 °C and the formation of the Pd_2_Ga phase is completed at around 500 °C.^[^ ^9^, ^29^ ^]^ Overall, the disparity in the area under peaks A2 and A3 in the reduction profiles of both Pd/SiO_2_ and Pd_2_Ga/SiO_2_ catalysts, along with the appearance of an additional peak at higher temperature, clearly indicate that the inclusion of Ga affects the reduction of PdO due to alloy formation and induces different extent of metal‐support interaction compared to Pd/SiO_2_.

### 2.3 CO_2_ Temperature‐Programmed Desorption (CO_2_‐TPD)

**Figure 4:**
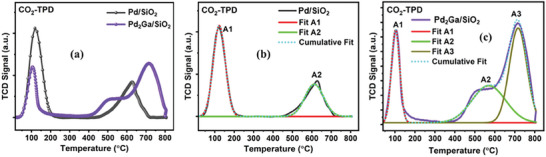
a) CO_2_‐TPD profiles of Pd/SiO_2_ and Pd_2_Ga/SiO_2_ catalysts. Deconvoluted profile for b) Pd/SiO_2_ and c) Pd_2_Ga/SiO_2_.

The surface affinity of the catalyst with CO_2_ present in the reaction feed plays a vital role in determining the catalytic activity/selectivity in CO_2_ conversion to methanol. CO_2_‐TPD experiments were conducted on Pd/SiO_2_ and Pd_2_Ga/SiO_2_ catalysts to understand the strength of Lewis basic sites and examine the impact of Ga addition on the strength of these sites (**Figure** **4**). Desorption peaks observed at temperatures below 250 °C (*T*_w_), between 250 and 500 °C (*T*_m_), and above 500 °C (*T*_s_) correspond to weak (w), moderate (m), and strong (s) basic sites, respectively.^[^ ^30^ ^]^

Both Pd‐based catalysts exhibit weak and strong basic sites, although the intensities of these peaks differ noticeably. The Pd/SiO_2_ catalyst exhibited a prominent peak at 122 °C and a moderately intense peak at 612 °C (Figure 4b). The calculated peak area ratio of weak basic sites (peak A1) to strong basic sites (peak A2) for Pd/SiO_2_ was found to be 1.82. In contrast, the Pd_2_Ga/SiO_2_ catalyst exhibited a broad and strong peak at 716 °C, with a shoulder peak at 498 °C (Figure 4c). The intensity of the peak observed at 105 °C, which corresponds to the region of weak basic sites was less than that of Pd/SiO_2_, giving a peak area ratio (A1/A2) of 0.26. These results indicate that the incorporation of Ga into the Pd catalyst significantly enhances the abundance of Lewis basic sites. By calibrating the thermal conductivity detector (TCD) signal, the total amount of desorbed/adsorbed CO_2_ can be determined, providing insights into the total number of surface basic sites on the studied oxides. In this regard, the order of total surface basic sites for both catalysts is as follows: Pd/SiO_2_ (0.48 mmol g^−1^) < Pd_2_Ga/SiO_2_ (0.68 mmol g^−1^). These sites are anticipated to play a favorable role in stabilizing intermediate species formed during the reaction, particularly as gallium‐rich sites facilitate methanol synthesis.^[^ ^8a^ ^]^

### 2.4 Microkinetic Model

The activation of CO_2_ and its interaction with the Pd and Pd alloy surfaces are crucial for the selective formation of methanol while inhibiting the reverse water gas shift reaction (RWGS) reaction. While Pd has been reported to poorly interact with CO_2_, the activity for methanol has increased significantly by adding another element in both alloyed and non‐alloyed forms.^[^ ^31^ ^]^ The role of intermetallics and their effect on the reaction intermediate energies is preferable for an efficient catalyst design. Kinetic modeling studies have been extensively reported for Cu‐based catalysts based on different mechanisms involving CO_2_ dissociation, HCOO, and COOH route, whereas the H_2_COO formation has mostly been found to be the rds for methanol synthesis (from both CO_2_ and CO_2_/CO mixture).^[^ ^32^ ^]^

**Figure 5:**
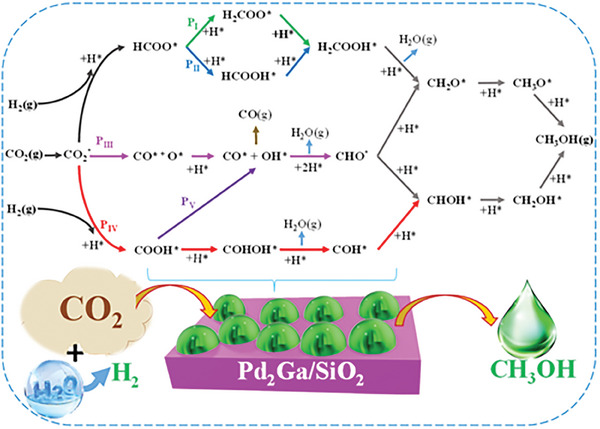
Schematic paths of CO_2_ hydrogenation to methanol over the Pd_2_Ga/SiO_2_ for RPA.

Most of the reported kinetic models for methanol synthesis derived using silica‐supported noble‐metal catalysts are based on the Langmuir‐Hinshelwood‐Hougen‐Watson (LH‐HW) method.^[^ ^23^, ^33^ ^]^ In this study, reactant adsorption, product desorption, and the reaction are based on intermetallic Pd_2_Ga, a single‐site mechanism. Fiordaliso et al.^[^ ^9a^ ^]^ observed that the Pd_2_Ga catalyst exhibited superior performance in methanol synthesis compared to Cu/ZnO/Al_2_O_3_ when operated under conditions of 1 bar pressure and temperatures ranging from 160 °C to 250 °C. Additionally, various mechanistic parameters acquired through DFT for the hydrogenation of carbon dioxide to CH_3_OH over Pd_2_Ga(010) sites were employed to investigate the reaction mechanism.^[^ ^34^ ^]^ The proposed mechanism consists of five pathways (**Figure** **5**), including three major pathways that start from COOH*, CO*, and HCOO* intermediates and lead to CH_3_OH, passing through various possible transition states. Correspondingly, the rate constant ($k\;=\;\frac{k_{B}T}{h}e^{-\frac{G_{a}}{RT}}$) for micro‐kinetics were obtained by Wu et al.^[^ ^34^ ^]^ in the same temperature range, where *R*, *T*, *h*, *k*_B_, and *G*_a_ represent the gas constant, absolute temperature, Planck constant, Boltzmann constant, and free energy barrier, respectively.

**Figure 6:**
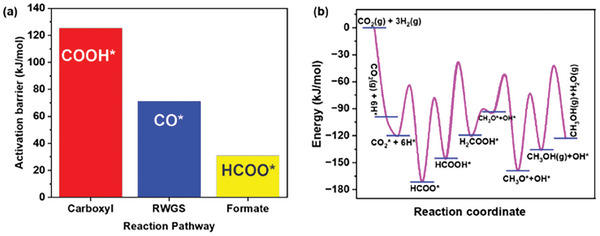
a) Most favorable pathway for methanol synthesis and b) starting pathway activation barrier.

The proposed elementary reaction steps, along with their respective kinetic parameters constituting the methanol synthesis mechanism and their corresponding forward rate expressions, are given in **Table** **1**. The activation energy and the rate constant for the forward reaction steps are represented by $\vec{E_{i}}$ and *k_i_*, respectively. After CO_2_ adsorption and H_2_ dissociative adsorption, the subsequent steps involve either the dissociation of CO_2_ into CO* and O* or the hydrogenation of CO_2_ to form HCOO* and COOH*. The overall general mechanism of the reaction comprises the three initial intermediates, undergoing successive hydrogenation through various possible reaction pathways, including dead‐end routes leading to the formation of CO and methanol. As given in Table 1, the activation barriers of the primary steps, S3(COOH*), S10(CO*), and S15(HCOO*), leading to three major pathways for methanol synthesis, are in the order of E_S3_ > E_S10_ > E_S15_ (**Figure** **6**a). Therefore, the reaction will proceed through the formate pathway over the intermetallic Pd_2_Ga catalyst. Furthermore, during subsequent hydrogenation, the HCOO* intermediate undergoes pathways P_I_ and P_II_, forming H_2_COO* and HCOOH*, respectively. However, the activation barrier to form HCOOH (93.46 kJ mol^−1^) is lower than that for H_2_COO* formation (139.71 kJ mol^−1^). Consequently, methanol formation will occur through the formate pathway via P_II_. Hence, the most probable pathway for methanol formation involves the reaction sequence S1 → S2 → S15 → S17 → S19 → S20 → S21 → S22 → S24. The potential energy diagram for the favorable methanol formation pathway from the direct hydrogenation of CO_2_ at 1/12 monolayer coverage is given in Figure 6b.

**Table 1:** Single‐site microkinetic model for CO_2_ hydrogenation to methanol over Pd_2_Ga/SiO_2_ catalyst.^[^ ^34^ ^]^

S. no.	Elementary reaction	$\vec{E_{i}}$ [kJ mol^−1^]	*k*_i_ (1/bar*^n^* s)^a)^	Forward rate, *r*_i_
1	H_2_(g) + 2* ↔ H* + H*	67.3	1.42×10^6^	$k_{1}P_{H_{2}}C_{s}^{2}$
2	CO_2_(g) + * ↔ CO_2_*	47.01	1×100	$k_{2}P_{CO_{2}}C_{s}$
3	CO_2_* + H* ↔ COOH* + *	125.26	8.22×10^−4^	$k_{3}K_{2}P_{CO_{2}}{\left(K_{1}P_{H_{2}}\right)}^{\frac{1}{2}}C_{s}^{2}$
4	COOH* + * ↔ CO* + OH*	25.05	2.412×10^7^	$k_{4}K_{2}K_{3}P_{CO_{2}}{\left(K_{1}P_{H_{2}}\right)}^{\frac{1}{2}}C_{s}^{2}$
5	COOH* + H* ↔ COHOH* + *	110.80	2.66×10^−2^	$k_{5}K_{1}K_{2}K_{3}P_{CO_{2}}P_{H_{2}}C_{s}^{2}$
6	COHOH* + * ↔ COH* + OH*	114.66	1.05×10^−3^	$k_{6}K_{1}K_{2}K_{3}K_{5}P_{CO_{2}}P_{H_{2}}C_{s}^{2}$
7	COH* + H* ↔ CHOH* + *	87.68	6.92×10^8^	$k_{7}K_{1}^{2}K_{2}K_{3}K_{5}K_{6}K_{24}P_{CO_{2}}P_{H_{2}}^{2}P_{H_{2}O}^{-1}C_{s}^{2}$
8	CHOH* + H* ↔ CH_2_OH* + *	56.85	1.11×10^4^	$k_{8}K_{1}^{2.5}K_{2}K_{3}K_{5}K_{6}K_{7}K_{24}P_{CO_{2}}P_{H_{2}}^{2.5}P_{H_{2}O}^{-1}C_{s}^{2}$
9	CH_2_OH* + H* ↔ CH_3_OH(g) + 2*	51.07	4.63×10^4^	$k_{9}K_{1}^{3}K_{2}K_{3}K_{5}K_{6}K_{7}K_{8}K_{24}P_{CO_{2}}P_{H_{2}}^{3}P_{H_{2}O}^{-1}C_{s}^{2}$
10	CO_2_* + * ↔ CO* + O*	71.30	3.56×10^6^	$k_{10}K_{2}P_{CO_{2}}C_{s}^{2}$
11	CO* ↔ CO(g) + *	56.21	1.00×10^0^	$k_{11}K_{11}^{-1}P_{\mathrm{CO}}C_{s}$
12	CO* + H* ↔ CHO* + *	116.59	6.62×10^−3^	$k_{12}K_{11}P_{\mathrm{CO}}{\left(K_{1}P_{H_{2}}\right)}^{\frac{1}{2}}C_{s}^{2}$
13	CHO*+ H* ↔ CH_2_O* + *	53.96	2.31×10^−5^	$k_{13}K_{1}K_{11}K_{12}P_{\mathrm{CO}}P_{H_{2}}C_{s}^{2}$
14	CHO*+ H* ↔ CHOH* + *	94.42	1.37×10^−7^	$k_{14}K_{1}K_{11}K_{12}P_{\mathrm{CO}}P_{H_{2}}C_{s}^{2}$
15	CO_2_* + H* ↔ HCOO* + *	30.83	6.01×10^7^	$k_{15}K_{2}P_{CO_{2}}{\left(K_{1}P_{H_{2}}\right)}^{\frac{1}{2}}C_{s}^{2}$
16	HCOO* + H* ↔ H_2_COO* + *	139.71	2.54×10^−6^	$k_{16}K_{1}K_{2}K_{15}P_{CO_{2}}P_{H_{2}}C_{s}^{2}$
17	HCOO* + H* ↔ HCOOH* + *	93.46	1.72×10^8^	$k_{17}K_{1}K_{2}K_{15}P_{CO_{2}}P_{H_{2}}C_{s}^{2}$
18	H_2_COO* + H* ↔ H_2_COOH* + *	99.24	4.29×10^−3^	$k_{18}K_{1}^{1.5}K_{2}K_{15}K_{16}P_{CO_{2}}P_{H_{2}}^{1.5}C_{s}^{2}$
19	HCOOH* + H* → H_2_COOH* + *	106.95	6.72×10^−4^	$k_{19}K_{1}^{1.5}K_{2}K_{15}K_{17}P_{CO_{2}}P_{H_{2}}^{1.5}C_{s}^{2}$
20	H_2_COOH* + * ↔ CH_2_O* + OH*	26.02	1.92×10^7^	$k_{20}K_{1}^{1.5}K_{2}K_{15}K_{17}K_{19}P_{CO_{2}}P_{H_{2}}^{1.5}C_{s}^{2}$
21	CH_2_O*+ H* ↔ CH_3_O* + *	36.61	1.49×10^6^	$k_{21}K_{1}^{2}K_{2}K_{15}K_{17}K_{19}K_{24}P_{CO_{2}}P_{H_{2}}^{2}P_{H_{2}O}^{-1}C_{s}^{2}$
22	CH_3_O* + H* ↔ CH_3_OH(g) + 2*	86.72	8.73×10^−2^	$k_{22}K_{1}^{2.5}K_{2}K_{15}K_{17}K_{19}K_{21}K_{24}P_{CO_{2}}P_{H_{2}}^{2.5}P_{H_{2}O}^{-1}C_{s}^{2}$
23	O* + H* ↔ OH* + *	98.28	5.41×10^−3^	$k_{23}K_{10}K_{11}P_{CO_{2}}P_{\mathrm{CO}}^{-1}{\left(K_{1}P_{H_{2}}\right)}^{\frac{1}{2}}C_{s}^{2}$
24	OH* + H* ↔ H_2_O(g) + 2*	94.42	1.37×10^8^	$k_{24}K_{24}^{-1}P_{H_{2}O}C_{s}^{2}$

* Indicates a metallic Pd_2_Ga site;

^a)^for adsorption steps *n* = 1, for surface reaction and desorption steps *n* = 0.

Considering each step as the rds, the resulting specific rates can be expressed as provided in Table 1. These expressions involve the partial pressures of reactants and products (*P*_i_), the equilibrium constants (*K*_i_), and reaction rate constants (*k*_i_). The rate expressions along with their respective kinetic parameters are employed to estimate the resistance encountered in each reaction step and the concentration of various surface intermediates at varying temperatures. The utilization of RPA facilitated the formulation of the rate expression for methanol synthesis and the identification of the rds and the MARI in the reaction.^[^ ^35^ ^]^ The comprehensive procedure and terminology for deriving the rate expression have been documented in previous publications.^[^ ^36^ ^]^ The rate expressions for individual steps were derived by applying the LH‐HW principle^[^ ^35^ ^]^ and the step resistance was computed according to the procedure outlined in the methodology section. Furthermore, the expressions for the surface concentration of all the reaction intermediates are given in **Table** **2**.

**Table 2:** Surface concentration expressions of all reaction intermediates over the Pd_2_Ga active sites.

S. no.	Reaction intermediate (occupied sites)		Surface concentration
1	[H*]		${\left(K_{1}P_{H_{2}}\right)}^{\frac{1}{2}}$
2	[CO_2_*]		$K_{2}P_{CO_{2}}$
3	[COOH*]		$K_{3}K_{2}P_{CO_{2}}{\left(K_{1}P_{H_{2}}\right)}^{\frac{1}{2}}$
4	[CO*]		$K_{11}^{-1}P_{\mathrm{CO}}$
5	[O*]		$K_{2}K_{10}K_{11}P_{CO_{2}}P_{\mathrm{CO}}^{-1}$
6	[COHOH*]		$K_{1}K_{2}K_{3}K_{5}P_{CO_{2}}P_{H_{2}}$
7	[COH*]		$K_{1}^{1.5}K_{2}K_{3}K_{5}K_{6}K_{24}P_{CO_{2}}P_{H_{2}}^{1.5}P_{H_{2}O}^{-1}$
8	[CHOH*]		$K_{7}K_{1}^{2}K_{2}K_{3}K_{5}K_{6}K_{24}P_{CO_{2}}P_{H_{2}}^{2}P_{H_{2}O}^{-1}$
9	[CH_2_OH*]		$K_{1}^{2.5}K_{2}K_{3}K_{5}K_{6}K_{7}K_{8}K_{24}P_{CO_{2}}P_{H_{2}}^{2.5}P_{H_{2}O}^{-1}$
10	[OH*]		$K_{24}^{-1}K_{1}^{-0.5}P_{H_{2}}^{-0.5}P_{H_{2}O}$
11	[CHO*]	$K_{11}K_{12}P_{\mathrm{CO}}{\left(K_{1}P_{H_{2}}\right)}^{\frac{1}{2}}$	
12	[HCOO*]	$K_{2}K_{15}P_{CO_{2}}{\left(K_{1}P_{H_{2}}\right)}^{\frac{1}{2}}$	
13	[H_2_COO*]	$K_{1}K_{2}K_{15}K_{16}P_{CO_{2}}P_{H_{2}}$	
14	[HCOOH*]	$K_{1}K_{2}K_{15}K_{17}P_{CO_{2}}P_{H_{2}}$	
15	[H_2_COOH*]	$K_{1}^{1.5}K_{2}K_{15}K_{16}K_{18}P_{CO_{2}}P_{H_{2}}^{1.5}$	
16	[CH_2_O*]	$K_{1}^{2}K_{2}K_{15}K_{17}K_{19}K_{24}P_{H_{2}}^{2}P_{H_{2}O}^{-1}$	
17	[CH_3_O*]	$K_{1}^{2}K_{2}K_{15}K_{17}K_{19}K_{21}K_{24}P_{CO_{2}}P_{H_{2}}^{2}P_{H_{2}O}^{-1}$	

Equation 1 balances the site for active sites involved summing up the adsorption terms of adsorbed intermediate species. The *C*_T_ and *C*_S_ represent the fraction of total sites and vacant catalyst surface active sites, respectively, whereas the occupied sites are represented by different reaction intermediates.

(1) $$C_{T}=1=\left(\begin{array}{c} C_{S}+\left[H*\right]+\left[CO_{2}*\right]+\left[\mathrm{COOH}*\right]+\left[\mathrm{CO}*\right]+\left[O*\right]+\left[\mathrm{COHOH}*\right]+\\ \left[\mathrm{COH}*\right]+\left[\mathrm{CHOH}*\right]+\left[CH_{2}\mathrm{OH}*\right]+\left[\mathrm{OH}*\right]+\left[\mathrm{CHO}*\right]+\left[\mathrm{HCOO}*\right]\\ +\left[H_{2}\mathrm{COO}*\right]+\left[\mathrm{HCOOH}*\right]+\left[H_{2}\mathrm{COOH}*\right]+\left[CH_{2}O*\right]+\left[CH_{3}O*\right] \end{array}\right)$$

The concentration of adsorbed species can be replaced and expressed in terms of the *K*_i_ and *P*_i_ as shown in Equation (2).

(2) $$\begin{array}{ccc} C_{S} & = & 1/[1+{\left(K_{1}P_{H_{2}}\right)}^{\frac{1}{2}}+K_{2}P_{CO_{2}}+K_{3}K_{2}P_{CO_{2}}{\left(K_{1}P_{H_{2}}\right)}^{\frac{1}{2}}\\ & & +\;K_{11}^{-1}P_{\mathrm{CO}}+K_{2}K_{10}K_{11}P_{CO_{2}}P_{\mathrm{CO}}^{-1}+K_{1}K_{2}K_{3}K_{5}P_{CO_{2}}P_{H_{2}}\\ & & +\;K_{1}^{1.5}K_{2}K_{3}K_{5}K_{6}K_{24}P_{CO_{2}}P_{H_{2}}^{1.5}P_{H_{2}O}^{-1}\\ & & +\;K_{7}K_{1}^{2}K_{2}K_{3}K_{5}K_{6}K_{24}P_{CO_{2}}P_{H_{2}}^{2}P_{H_{2}O}^{-1}\\ & & +K_{1}^{2.5}K_{2}K_{3}K_{5}K_{6}K_{7}K_{8}K_{24}P_{CO_{2}}P_{H_{2}}^{2.5}P_{H_{2}O}^{-1}+K_{24}^{-1}K_{1}^{-0.5}P_{H_{2}}^{-0.5}P_{H_{2}O}\\ & & +\;K_{11}K_{12}P_{\mathrm{CO}}{\left(K_{1}P_{H_{2}}\right)}^{1/2}+K_{2}K_{15}P_{CO_{2}}{\left(K_{1}P_{H_{2}}\right)}^{1/2}\\ & & +\;K_{1}K_{2}K_{15}K_{16}P_{CO_{2}}P_{H_{2}}+K_{1}K_{2}K_{15}K_{17}P_{CO_{2}}P_{H_{2}}\\ & & +\;K_{1}^{1.5}K_{2}K_{15}K_{16}K_{18}P_{CO_{2}}P_{H_{2}}^{1.5}+K_{1}^{2}K_{2}K_{15}K_{17}K_{19}K_{24}P_{H_{2}}^{2}P_{H_{2}O}^{-1}\\ & & +\;K_{1}^{2}K_{2}K_{15}K_{17}K_{19}K_{21}K_{24}P_{CO_{2}}P_{H_{2}}^{2}P_{H_{2}O}^{-1}] \end{array}$$

**Figure 7:**
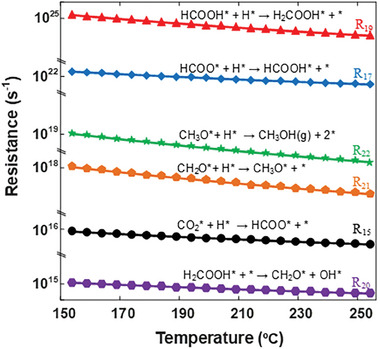
Reaction step resistance from RPA at different temperatures over Pd_2_Ga/SiO_2_.

Equation 2 is used to expand the description of the rate expression of different elementary reaction steps provided in Table 1. The reaction rates and equilibrium values within the experimental range of pressure and temperature provide insight into step resistances. The step resistances for methanol synthesis are calculated by taking the inverse of the rate expressions from Table 1. The calculated step resistance values at various temperatures within the experimental range (Table S3, Supporting Information) are compared and presented in **Figure** **7** to identify the step with the maximum resistance, commonly referred to as the rds. As shown in Figure 7, step 19, involving the hydrogenation of HCOOH* to the H_2_COOH* intermediate, exhibits the highest resistance in the context of methanol formation. These results suggest that step 19 has the slowest rate, influencing the overall methanol synthesis. The rate law based on the rds (step 19) for methanol synthesis over the Pd_2_Ga catalyst is expressed in Equations (3) and (4). The results obtained from the RPA align with the mechanisms reported by Kowalec et al.^[^ ^37^ ^]^ for all three facets, (111), (100), and (110) of Pd, as well as Yao et al.^[^ ^38^ ^]^ for Cu_3_Zn alloys.

(3) $$\mathrm{rat}e_{CH_{3}\mathrm{OH}}=r_{19}\;=k_{19}\;\left[\mathrm{HCOO}H^{*}\right]\left[H^{*}\right]-k_{-19}\left[H_{2}\mathrm{COO}H^{*}\right]C_{s}^{2}$$

(4) rCH3OH=k19K11.5PH21.5PCH3OHPH2OK19K20K21K24K13PH23K2K15K17PCO2−PCH3OHPH2OK19K20K21K24K13PH23Cs2

Furthermore, to simplify the rate expression, it is essential to determine MARI over the catalyst surface. In this case, MARI investigation includes the estimation of fractional surface coverage for all intermediates, including CO_2_*, CO*, H*, OH*, and HCOO*.

**Figure 8:**
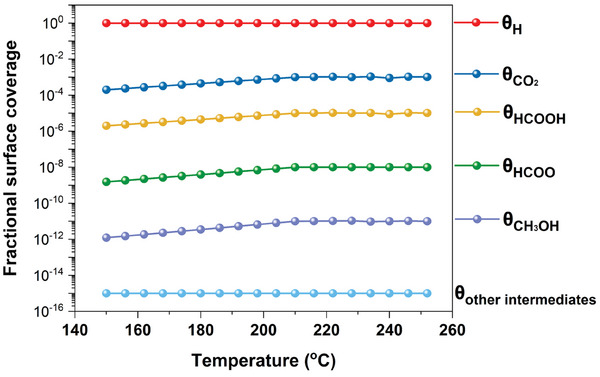
Fractional surface coverage of adsorbed/intermediate species on the Pd_2_Ga catalyst (Supporting Information).

**Figure** **8** results indicate that adsorbed reactant species comprise the highest fractional surface coverage among all the species. This predominant coverage of H‐species on the surface of the catalyst may be attributed to the spontaneous dissociation of H_2_.

The relatively lower surface coverage of the other reaction intermediates and products can be ascribed to their short‐lived presence and rapid desorption. Consequently, Equation (5) can be simplified to Equation (6), resulting in the final expression for the rate of CH_3_OH formation from CO_2_/H_2_ over the Pd_2_Ga/SiO_2_ catalyst. The Pd_2_Ga catalyst generates CO as a byproduct during the CO_2_ hydrogenation process, while displaying a selectivity as high as 96% for methanol in experimental studies (Table S2, Supporting Information).

(5) $$r_{CH_{3}\mathrm{OH}}=\frac{k_{19}{K_{1}}^{1.5}{P_{H_{2}}}^{1.5}\left[K_{2}K_{15}K_{17}P_{CO_{2}}-\frac{P_{CH_{3}\mathrm{OH}}P_{H_{2}O}}{K_{19}K_{20}K_{21\;}K_{24}{K_{1}}^{3}{P_{H_{2}}}^{3}}\right]}{{\left(1+K_{1}P_{CO_{2}}+\sqrt{K_{2}P_{H_{2}}}\right)}^{2}}\;$$

(6) $$r_{\mathrm{RWGS}}=\frac{k_{10}\left[K_{2}P_{CO_{2}}-\frac{P_{\mathrm{CO}}P_{H_{2}O}}{K_{10}K_{11}K_{23\;}K_{24}K_{1}P_{H_{2}}}\right]}{{\left(1+K_{1}P_{CO_{2}}+\;\sqrt{K_{2}P_{H_{2}}}\right)}^{2}}\;$$

There are two distinct pathways for CO production: either through the dissociation of CO_2_ or via the decomposition of COOH. Notably, the latter pathway has a higher starting activation energy barrier (Step 3, $\vec{E}$= 125.26 kJ mol^−1^). As a result, the RWGS pathway, where CO_2_ is dissociated (Step 10, $\vec{E}$= 70.30 kJ mol^−1^) and serves as the rds, is the favorable pathway for CO production. It indicates that the pathway terminates immediately after the formation of CO. Based on the rds (Step 10: CO_2_* + * → CO* + O*), MARI, and LH‐HW method, the formation rate of CO via the RWGS reaction is derived and given in Equation (6).

### 2.5 Microkinetic Model Verification: Parameter Estimation and Curve Fitting

For comparison with the actual reaction of the catalyst surface and model verification, experimental kinetic analysis was performed in a differential plug flow fixed‐bed reactor. Gradual variations were applied to the weight hourly space velocity (WHSV), T, H_2_/CO_2_ ratio, and P to determine CO_2_ conversion, selectivity, and the formation of methanol and CO (Table S2, Supporting Information). All kinetic data were obtained in a mass transfer‐free regime by evaluating the internal and external mass transfer limitations.^[^ ^19^ ^]^ Furthermore, the Pd_2_Ga/SiO_2_ intermetallic was found to be active for methanol synthesis, whereas no methanol formation was observed over the Pd/SiO_2_ catalyst. The rate of methanol or CO formation (*r*_p_) was calculated using a differential equation for the isothermal reactor design as per Equation (7).

(7) $$\left(\frac{W}{F_{CO_{2}}}\right)=\left(\frac{X_{CO_{2}}}{r_{p}}\right)\;$$

where *W* is the catalyst weight, $F_{CO_{2}}$ represents the molar flow rate, and $X_{CO_{2}}$ is the percent CO_2_ conversion.

**Figure 9:**
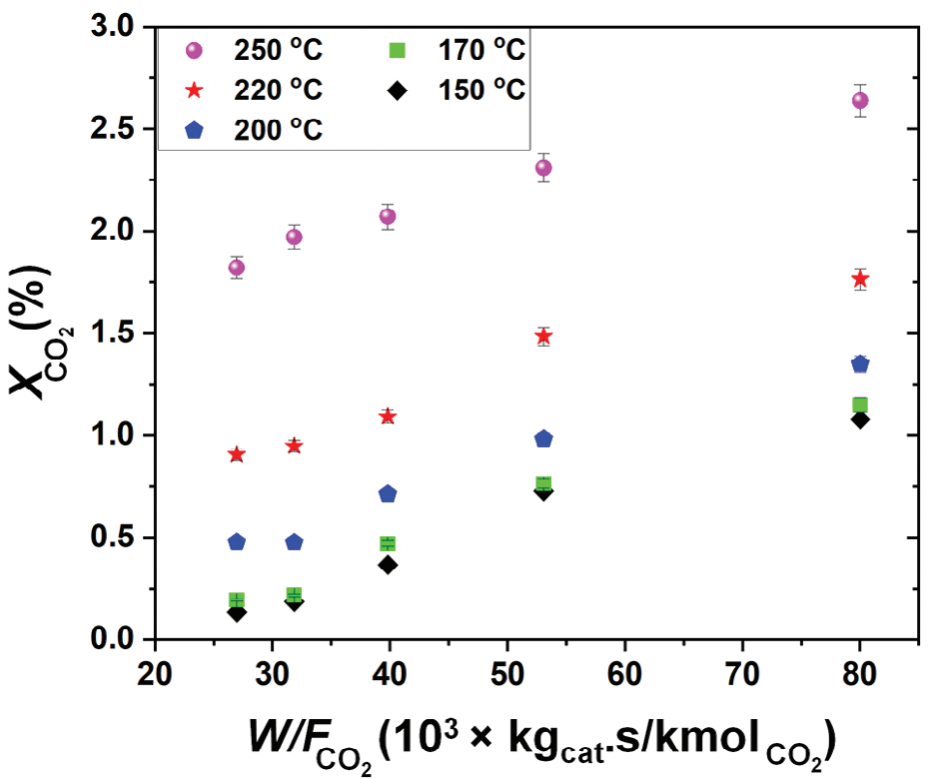
CO_2_ conversion over the reported Pd_2_Ga/SiO_2_ catalyst at 1 bar for different *W*/*F*_CO2_ conditions.

According to **Figure** **9**, the experimental conversion of CO_2_ followed a consistent path with a linear behavior of the $W/F_{CO_{2}}$ term under isothermal conditions. The linear behavior and the constant rate of the reaction in isothermal CO_2_ conversion curves indicate the differential characteristics of the reaction.

Herein, the reaction is considered to follow the single‐site model (competitive adsorption). The denominators of the rate equations for methanol synthesis (Equation (5)) and RWGS (Equation (6)) reactions demonstrate similarity. For parameter estimation, the Arrhenius and Van't Hoff equations ($k_{i}=\;A_{i}e^{\left(-B_{i}/RT\right)}$) were employed to estimate the constants of reaction rate and equilibrium, with careful consideration of statistical significance and physical meaning.^[^ ^39^ ^]^ Here, *B*_i_ represents ∆*E*_i_ and ∆*H*_i_ in the Arrhenius and Van't Hoff equations, respectively. To address computational complexities ascending from the strong relationship among frequency factors and activation energies, the model constants were formulated in a parameterized manner as depicted by the equation ($k_{i}=\;A_{i}e^{\left[B_{i}\left(\frac{1}{T_{\mathrm{av}}}-\frac{1}{T}\right)\right]}$). The AspenPlus software package was employed to estimate the *K*_i_ for methanol synthesis and the RWGS reaction via Equations (8) and (9), respectively.

(8) $$\mathrm{lo}g_{10}\;K_{CH_{3}\mathrm{OH}}=\frac{2936.62998}{T}\;-10.22557$$

(9) $$\mathrm{lo}g_{10}\;K_{\mathrm{RWGS}}=\frac{2097.57404}{T}\;-2.06011$$

The simulation of data and the estimation of parameters were carried out using a nonlinear regression tool within MATLAB, employing the lsqnonlin technique, which utilizes the Levenberg‐Marquardt method. The optimization aimed to minimize the objective function outlined in Equation (10) to enhance the accuracy of the estimated kinetic model parameters.

(10) $$f\;=\sum_{i=1}^{n}{\left[{\left(r_{\mathrm{model}}\right)}_{i}\;-\;{\left(r_{\exp}\right)}_{i}\right]}^{2}$$

The symbols *r*_model_ and *r*_exp_ denote the computed (from the kinetic model) and observed rates in CH_3_OH synthesis, respectively. The total count of experimental runs is denoted by “n.” Statistical significance across diverse iterations and the optimal curve fit was evaluated using various methods, including the coefficient of determination (*R* ^2^), minimum acceptable rate of return (MARR), and root mean square error (RMSE). Detailed information and formulas for these mathematical parameters can be explored elsewhere.^[^ ^19^ ^]^ The results of process optimization and the values of kinetic parameters are displayed in **Table** **3**. The optimization outcomes are not only physically meaningful but also statistically significant, meeting the specified criteria.^[^ ^40^ ^]^

**Table 3:** Estimated kinetic model parameters and statistical results.

Kinetic model parameter	Value
*k*_methanol_	$3.68\times 10^{6}e^{\left(\frac{-61.532}{RT}\right)}$
*E*_methanol_	61.52
*k*_RWGS_	$3.81\times 10^{5}e^{\left(\frac{-109.861}{RT}\right)}$
*E*_RWGS_	109.91
$K_{CO_{2}}$	$2.51\times 10^{-9}e^{\left(\frac{86.021}{RT}\right)}$
$K_{H_{2}}$	$5.12\times 10^{5}e^{\left(\frac{79.461}{RT}\right)}$
*R* ^2^	0.99
%MARR	0.69
RMSE	1.59

**Figure 10:**
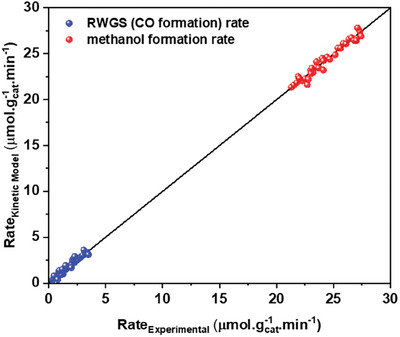
Parity plot for CH_3_OH synthesis and RWGS reaction based on the derived kinetic models and obtained experimental data (Table S2, Supporting Information).

The statistical analysis and the estimated parameters indicate that methanol synthesis over intermetallic Pd_2_Ga follows a single‐site mechanism. The activation energy of 61.52 kJ mol^−1^ for the methanol synthesis was found to be consistent with different reported catalysts. The activation energy is much lower than reported over various Cu catalysts like PdCuZn/SiC (116.6 kJ mol^−1^),^[^ ^21^ ^]^ and CuO/ZnO/Al_2_O_3_ (104.6 kJ mol^−1^)^[^ ^41^ ^]^ whereas the same is higher than reported over Cu/ZnO/ZrO_2_/Al_2_O_3_/SiO_2_ (32.1 kJ mol^−1^).^[^ ^39^ ^]^ Aside from the lumping of parameters (the product of *k*_i_ and *K*_eq_), the difference in the activation energy values can be ascribed to factors such as the mode of adsorption, type of catalyst, and the presence of different crystalline planes in a catalyst. Furthermore, the difference in *E*_a_ values between experimental data and DFT values can be attributed to the presence of multiple sites (edge, step, kink, or corner) and planes in a catalyst, whereas the DFT study has been carried out over a single plane. The resulting overall activation energy was found to be within a similar range as reported for Cu‐Zn‐Al (65.2 kJ mol^−1^)^[^ ^33c^ ^]^ and Cu/ZnO/Al_2_O_3_ (68.3 kJ mol^−1^)^[^ ^42^ ^]^ catalysts. The rates of methanol and CO formation, calculated from their respective derived kinetic models, are compared with the experimental reaction rates, as illustrated in **Figure** **10**. The strong agreement with an R^2^ value of 0.99 between the model‐calculated and experimental values provides additional support for the developed mechanism. These results highlight the accuracy and robustness of the RPA methodology employed in investigating reaction mechanisms, including the identification of rds and MARI, and subsequently, in developing the kinetic model.

## 3 Conclusions

A novel combined experimental and theoretical investigation into the kinetics of methanol synthesis from CO_2_ hydrogenation via the RPA methodology over intermetallic Pd_2_Ga/SiO_2_ is presented. The intermetallic Pd_2_Ga was synthesized using the incipient wetness impregnation method, and its physiochemical properties were investigated using XRD, HR‐TEM, H_2_‐TPR, and CO_2_‐TPD. The formation of a pure‐phase Pd_2_Ga catalyst (having a crystal size of 5.73 nm) was confirmed by both XRD and SAED results. H_2_‐TPR analysis revealed that the introduction of Ga in the Pd catalyst (supported over SiO_2_) enhanced the metal–support interaction, resulting in stability against sintering during the exothermic methanol synthesis reaction. Furthermore, the increase in the number of basic sites by the alloying of Ga with Pd was revealed in CO_2_‐TPD. Comparatively high basicity in Pd_2_Ga/SiO_2_ catalyst compared to Pd/SiO_2_ indicated the high concentration of adsorbed CO_2_ on the catalyst surface (Pd/SiO_2_ (0.48 mmol g^−1^) < PdGa/SiO_2_ (0.68 mmol g^−1^), which, in turn, is useful for methanol synthesis. The direct hydrogenation of CO_2_ over the Pd_2_Ga/SiO_2_ catalyst resulted in high selectivity to methanol (96% at *P* = 1 bar), whereas the Pd/SiO_2_ was found inactive for methanol synthesis. The results indicate the critical importance of the intermetallic Pd_2_Ga phase. From the kinetic study using RPA methodology, methanol synthesis was found to follow the formate route with HCOOH* hydrogenation to H_2_COOH* as the rds, and CO_2_* and H* were found to be the MARI. Whereas the dissociation of CO_2_ into CO* and O* was found to be the rds for the RWGS reaction. Subsequently, kinetic models for methanol synthesis and the RWGS reaction were derived and validated, and they were found to fit the experimental data. Parameter estimation revealed that methanol synthesis and RWGS have an activation energy of 61.52 and 109.91 kJ mol^−1^, respectively, which are lower than those reported over many catalysts. The mechanism study and kinetic modeling presented in this paper, utilizing a wide range of both theoretical and experimental data, can be regarded as a comprehensive approach to achieving highly reliable results with accuracy and robustness. The procedure and results apply not only to other kinetic studies but also to the development of highly selective catalysts, especially for atmospheric CO_2_ conversion reactions.

## 4 Experimental Section

### Catalyst Preparation and Characterization

The incipient wetness impregnation method using metal precursors, Ga(NO_3_)_3_.*x*H_2_O and Pd(NO_3_)_3_.6H_2_O with SiO_2_ as the support, was used to synthesize the Pd_2_Ga/SiO_2_ catalyst.^[^ ^9b^ ^]^ In summary, a solution containing appropriate amounts of palladium and gallium nitrate hydrate (having a molar ratio of Pd:Ga = 2:1) with 99.99% purity was prepared in a 4 M nitric acid solution and impregnated onto silica support. The resulting catalyst was dried for 24 h at 120 °C and then calcined for 4 h at 260 °C. Subsequently, the catalyst bed temperature was raised to 600 °C at a rate of 5 °C min^−1^ in pure argon flow and then reduced in the 10% hydrogen in argon gas mixture for 2 h. Similarly, for comparison purposes, a silica‐supported Pd catalyst was synthesized following the same procedure by using the appropriate quantities of precursors. Essential properties of the Pd_2_Ga/SiO_2_ encompassing the microstructure and degree of crystallinity, surface and bulk reducibility, as well as alkalinity titration were determined using various techniques like p‐XRD, bright‐field HR‐TEM, and SAED, H_2_‐TPR, and CO_2_‐TPD, respectively. The details of equipment and measurement procedures are reported elsewhere.^[^ ^30^ ^]^

### Catalytic Studies

The performance of the Pd_2_Ga/SiO_2_ and Pd/SiO_2_ catalysts was assessed in an Effi PID fixed bed automatic reactor made of 0.45–0.50 mm particles, with a bed volume of 1.13 cm^3^.

The reaction was conducted at *P* = 1 bar and different conditions of H_2_/CO_2_ ratio, temperature (*T*), and WHSV. The product analysis was carried out using gas chromatography, Nucon‐5700 with carbosphere and Porapak‐Q columns linked to a thermal conductivity detector (TCD) and a flame ionization detector (FID) for detecting CO_2_/CO and methanol, respectively. To ensure a plug flow environment, the catalyst fixed‐bed height (*L*), size of the catalyst granules (*d*_p_), and diameter of the reactor (*D*) were maintained at *L*/*d*_p_ ≥ 50 and *D*/*d*_p_ ≥ 1.^[^ ^43^ ^]^ Equations (11–, 14) were employed for the computation of the C‐balance (*C*_balance_), CO_2_ conversion ($X_{CO_{2}}$), and selectivity to CH_3_OH ($S_{CH_{3}\mathrm{OH}}$) and CO (*S*_CO_).

(11) $$C_{\mathrm{balance}}=\frac{F_{CH_{3}OH_{\mathrm{out}}}+F_{CO_{\mathrm{out}}}+F_{CO_{2,\mathrm{out}}}}{F_{CO_{2,in}}}\;$$

(12) $$X_{CO_{2}}=\frac{F_{CO_{2,\mathrm{in}}}-F_{CO_{2,\mathrm{out}}}}{F_{CO_{2,\mathrm{in}}}}\;$$

(13) $$S_{CH_{3}\mathrm{OH}}=\frac{F_{CH_{3}OH_{\mathrm{out}}}}{F_{CH_{3}OH_{\mathrm{out}}}+F_{CO_{\mathrm{out}}}}\;$$

(14) $$S_{\mathrm{CO}}=\frac{F_{CO_{\mathrm{out}}}}{F_{CH_{3}OH_{\mathrm{out}}}+F_{CO_{\mathrm{out}}}}\;$$

where *F*_i_ denotes the molar flow rate of the reactant and product species “i.”

The experimental data for verifying the kinetic model and estimating parameters were obtained in a mass transfer‐free regime, i.e., the catalytic reaction was free from internal and external mass transfer limitations. Additionally, the kinetic experiments were carried out in a heat transfer limitation‐free regime. The procedural steps of this work were adapted from the published studies on kinetic modeling.^[^ ^19^, ^23^ ^]^

### Reaction Path Analysis (RPA): Procedure

The computational RPA method is adopted to establish a comprehensive kinetic model for the CH_3_OH synthesis by studying the reaction kinetics from the micro‐ to macroscale. RPA can provide valuable insights into the underlying mechanisms of complex chemical reactions and guide the growth of more efficient and sustainable industrial processes. To examine the reaction mechanism, including the MARI and rds, and ensure the model's accuracy, the energy values for a wide range of elementary reaction steps forming different pathways were adapted from recently published DFT predictions by Wu et al.^[^ ^34^ ^]^ and included in the development of the various rate laws. In the process of RPA, briefly, calculations are analogous to Ohm's law, which is applied to an electrical circuit where the forward rate of reaction and its inverse are treated as current and resistance, respectively. To estimate step resistances, the LH‐HW method is employed, where each elementary step is treated as a rds (when the rest of the steps are in equilibrium), and the step resistances and overall resistance in a particular pathway are calculated in terms of partial pressure and rate constants. The total resistance from individual steps contributes to the overall kinetic resistance, with the elementary step possessing the highest resistance along the reaction pathway, determining the rds. Subsequently, the general reaction rate law derived is based on the resulting rds. Regarding the abundance of surface intermediates, the MARI investigation is executed based on derived expressions denoting individual surface concentrations of reaction intermediates on catalyst active sites. Finally, the kinetic parameter estimation and model verification are done using the experimental data obtained from a lab‐scale reactor.

## Conflict of Interest

The authors declare no conflicts of interests.

## Supporting information



Supporting Information

Supporting Information

## Data Availability

The data that support the findings of this study are available in the Supporting Information of this article.
