# Supplementary material for: Kinetic Insights into Methanol Synthesis from CO2 Hydrogenation at Atmospheric Pressure over Intermetallic Pd2Ga Catalyst
Source: Glob Chall. 2024 Sep 20;8(10):2400159. doi: 10.1002/gch2.202400159 (PMC11469785; doi:10.1002/gch2.202400159)
Supplement: Supplementary file 1 — Supporting Information [file GCH2-8-2400159-s002.docx]

**Kinetic Insights into Methanol Synthesis from CO_2_ Hydrogenation at Atmospheric Pressure over Intermetallic Pd_2_Ga Catalyst**

Kaisar Ahmad^1^*, Aasif Asharafbhai Dabbawala^2^, Kyriaki Polychronopoulou^2^, Dalaver Anjum^3^, Marko Gacesa^3^, Maguy Abi Jaoude^1^*

*^1^Center for Catalysis and Separations (CeCaS), Department of Chemistry, Khalifa University of Science and Technology, Abu Dhabi, PO Box 127788, United Arab Emirates*

*^2^Center for Catalysis and Separations (CeCaS), Department of Mechanical and Nuclear Engineering, Khalifa University of Science and Technology, PO Box 127788, Abu Dhabi, United Arab Emirates*

*^3^Center for Catalysis and Separations (CeCaS), Department of Physics, Khalifa University of Science and Technology, Abu Dhabi, PO Box 127788, United Arab Emirates*

**Corresponding authors:* [*kaisar.hajam@ku.ac.ae*](mailto:kaisar.hajam@ku.ac.ae)*;* [*maguy.abijaoude@ku.ac.ae*](mailto:maguy.abijaoude@ku.ac.ae)

**Supplementary information**

**Table S1.** Experimental reaction conditions [9b].

| **Parameter** | **Value** |
| --- | --- |
| Pd/Ga ratio | 2/1 |
| Catalyst surface area | 217.46 × 10^4^ (cm^2^/g) |
| Surface area SiO_2_-support | 250 × 10^4^ (cm^2^/g) |
| Catalyst bed volume | 0.113 (cm^3^) |
| Bed length | 10 (mm) |
| Volume/area of catalyst bed | 10.4 × 10^2^ (cm) |
| Reactor diameter | 6 (mm) |
| *W*/*F*_CO2_ | (26.96$\times$10^3^ to 80.02$\times$10^3^) $\mathrm{kg}_{cat}.s/\mathrm{kmol}_{\mathrm{CO}_{2}}$ |
| Feed ratio (H_2_/CO_2_) | 1/1 to 9/1 |
| Temperature | 150 to 250 ℃ |
| Pressure | 1 bar |

**Table S2** List of experiments for the kinetics at different reaction conditions.

| **Exp. No.** | **T**  (˚C) | ***W*/*F*_CO2_**  ($10^{3}\times\mathrm{kg}_{cat}.s/\mathrm{kmol}_{\mathrm{CO}_{2}}$) | **H_2_/CO_2_ ratio** | $\mathbf{X}_{\mathbf{CO}_{\mathbf{2}}}$  (%) | **R_MeOH_**  **(**$\mu mol.g_{\mathrm{cat}}^{-1}\min^{-1})$ | **S_MeOH_**  (%) |
| --- | --- | --- | --- | --- | --- | --- |
| 1 | 150 | 26.9600 | 3/1 | 0.13423 | 27.230 | 95.24 |
| 2 | 150 | 31.8494 | 3/1 | 0.18426 | 27.165 | 95.48 |
| 3 | 150 | 39.8118 | 3/1 | 0.36597 | 27.100 | 95.72 |
| 4 | 150 | 53.0824 | 3/1 | 0.72566 | 27.035 | 95.96 |
| 5 | 150 | 80.0200 | 3/1 | 1.07748 | 26.970 | 96.20 |
| 6 | 170 | 26.9600 | 3/1 | 0.19361 | 28.460 | 93.56 |
| 7 | 170 | 31.8494 | 3/1 | 0.21689 | 28.395 | 93.80 |
| 8 | 170 | 39.8118 | 3/1 | 0.46935 | 28.330 | 94.04 |
| 9 | 170 | 53.0824 | 3/1 | 0.76303 | 28.265 | 94.28 |
| 10 | 170 | 80.0200 | 3/1 | 1.14785 | 28.200 | 94.52 |
| 11 | 200 | 26.9600 | 3/1 | 0.47750 | 28.135 | 92.59 |
| 12 | 200 | 31.8494 | 3/1 | 0.47569 | 28.070 | 92.83 |
| 13 | 200 | 39.8118 | 3/1 | 0.71196 | 28.005 | 93.07 |
| 14 | 200 | 53.0824 | 3/1 | 0.98180 | 27.940 | 93.31 |
| 15 | 200 | 80.0200 | 3/1 | 1.34913 | 27.430 | 93.55 |
| 16 | 220 | 26.9600 | 3/1 | 0.90392 | 26.020 | 91.29 |
| 17 | 220 | 31.8494 | 3/1 | 0.94641 | 25.955 | 91.53 |
| 18 | 220 | 39.8118 | 3/1 | 1.09114 | 25.890 | 91.77 |
| 19 | 220 | 53.0824 | 3/1 | 1.48321 | 25.825 | 92.01 |
| 20 | 220 | 80.0200 | 3/1 | 1.76384 | 25.760 | 92.25 |
| 21 | 250 | 26.9600 | 3/1 | 1.82024 | 24.935 | 89.11 |
| 22 | 250 | 31.8494 | 3/1 | 1.96971 | 24.870 | 89.35 |
| 23 | 250 | 39.8118 | 3/1 | 2.07072 | 24.805 | 89.59 |
| 24 | 250 | 53.0824 | 3/1 | 2.30978 | 24.740 | 89.83 |
| 25 | 250 | 80.0200 | 3/1 | 2.63853 | 24.675 | 90.07 |
| 26 | 200 | 39.8118 | 1/1 | 0.65196 | 21.680 | 93.44 |
| 27 | 200 | 39.8118 | 2/1 | 0.68196 | 21.745 | 93.66 |
| 28 | 200 | 39.8118 | 3/1 | 0.71196 | 21.810 | 93.88 |
| 29 | 200 | 39.8118 | 4/1 | 0.73196 | 21.875 | 94.10 |
| 30 | 200 | 39.8118 | 5/1 | 0.75196 | 21.940 | 94.32 |
| 31 | 200 | 39.8118 | 6/1 | 0.77196 | 22.005 | 94.54 |
| 32 | 200 | 39.8118 | 7/1 | 0.79196 | 22.070 | 94.76 |
| 33 | 200 | 39.8118 | 8/1 | 0.81196 | 22.135 | 94.98 |
| 34 | 200 | 39.8118 | 9/1 | 0.83196 | 22.200 | 95.20 |

**Table S3.** Elementary reaction step resistance at different temperatures for the formate pathway.

| **Temperature**  **(℃)** | **Step resistance (1/rate)** | | | | | |
| --- | --- | --- | --- | --- | --- | --- |
|  | **19** | **17** | **22** | **21** | **15** | **20** |
| 150 | 5.29×10^25^ | 4.67×10^22^ | 1.09×10^19^ | 5.34×10^18^ | 1.97×10^16^ | 4.62×10^15^ |
| 160 | 5.29×10^25^ | 4.67×10^22^ | 1.09×10^19^ | 5.34×10^18^ | 1.97×10^16^ | 4.62×10^15^ |
| 170 | 5.29×10^25^ | 4.67×10^22^ | 1.09×10^19^ | 5.34×10^18^ | 1.97×10^16^ | 4.62×10^15^ |
| 180 | 6.13×10^25^ | 4.67×10^22^ | 1.09×10^19^ | 5.34×10^18^ | 1.97×10^16^ | 4.62×10^15^ |
| 190 | 6.13×10^25^ | 4.67×10^22^ | 1.09×10^19^ | 5.34×10^18^ | 1.97×10^16^ | 4.62×10^15^ |
| 200 | 6.13×10^25^ | 4.67×10^22^ | 1.09×10^19^ | 5.34×10^18^ | 1.97×10^16^ | 4.62×10^15^ |
| 210 | 6.13×10^25^ | 4.67×10^22^ | 1.09×10^19^ | 5.34×10^18^ | 1.97×10^16^ | 4.62×10^15^ |
| 220 | 6.13×10^25^ | 4.67×10^22^ | 1.09×10^19^ | 5.34×10^18^ | 1.97×10^16^ | 4.62×10^15^ |
| 230 | 6.13×10^25^ | 4.67×10^22^ | 1.09×10^19^ | 5.34×10^18^ | 1.97×10^16^ | 4.62×10^15^ |
| 240 | 6.13×10^25^ | 4.67×10^22^ | 1.09×10^19^ | 5.34×10^18^ | 1.97×10^16^ | 4.62×10^15^ |
| 250 | 6.13×10^25^ | 4.67×10^22^ | 1.09×10^19^ | 5.34×10^18^ | 1.97×10^16^ | 4.62×10^15^ |

# **Reference**

[9b] Ahmad K, Upadhyayula S. Influence of reduction temperature on the formation of intermetallic Pd_2_Ga phase and its catalytic activity in CO_2_ hydrogenation to methanol. Greenh Gases Sci Technol 2019;9:529–38. https://doi.org/10.1002/ghg.1872.
